# Black rice (*Oryza sativa L*.) extract attenuates hepatic steatosis in C57BL/6 J mice fed a high-fat diet via fatty acid oxidation

**DOI:** 10.1186/1743-7075-9-27

**Published:** 2012-03-30

**Authors:** Hwan-Hee Jang, Mi-Young Park, Heon-Woong Kim, Young-Min Lee, Kyung-A Hwang, Jae-Hak Park, Dong-Sik Park, Oran Kwon

**Affiliations:** 1 Functional Food & Nutrition Division, Department of Agrofood Resources,Rural Development Administration, Suwon, Republic of Korea; 2 College of Veterinary Medicine, Seoul National University, Seoul, Republic of Korea; 3 Department of Nutritional Science and Food Management, Ewha Woman's University, Seoul, Republic of Korea

**Keywords:** Black rice, Cyanidin-3-glucoside, Hepatic steatosis, High-fat diet, Fatty acid oxidation

## Abstract

**Background:**

Two major risk factors for the onset of fatty liver disease are excessive alcohol intake and obesity, the latter being associated with non-alcoholic fatty liver disease (NAFLD). The aim of this study was to examine the effects of black rice extract (BRE) on hepatic steatosis and insulin resistance in high-fat diet-fed mice, providing a model of NAFLD.

**Methods:**

Twenty-four mice were randomly divided into three groups (n = 8 in each group): normal fat diet (ND), high fat diet (HF), and high fat diet supplemented with 1% (w/w) BRE (HF +1% BRE). The experimental diets were fed for seven weeks.

**Results:**

A HF induced hepatic steatosis with significant increases in the serum levels of free fatty acids (FFAs), triglyceride (TG), total cholesterol (TC), and insulin. By contrast, supplementary BRE (10 g/kg of diet) included in the HF alleviated hepatic steatosis and significantly decreased serum TG and TC levels (p < 0.01 for both). Dietary BRE also increased expression of fatty acid metabolism-related genes, including carnitine palmitoyltransferase (CPT1A), acyl-CoA oxidase (ACO), cytochrome P450 (CYP4A10), and peroxisome proliferator activated receptor (PPAR)-α (p < 0.05 for all).

**Conclusions:**

Dietary BRE supplementation improved serum lipid profiles and significantly enhanced mRNA expression levels of fatty acid metabolism-related genes, primarily via β-oxidation and ω-oxidation in the liver. Taken together, these findings suggest that a BRE-supplemented diet could be useful in reducing the risks of hepatic steatosis and related disorders, including hyperlipidemia and hyperglycemia.

## Background

The liver is the primary fat-metabolizing organ. Normal cellular fatty acid homeostasis is the product of a balance between fatty acid uptake, utilization, and export from the liver, which is controlled by a complex transcriptional network that is attuned to meeting the energy requirements of cells while preventing excessive accumulation of fatty acids [1]. However, excessive dietary fat can result in increased free fatty acids (FFAs) levels in the blood, thereby amplifying the delivery of FFAs to the liver [2]. Thus, excessive consumption of dietary fats induces lipid accumulation in the liver and can eventually cause obesity. However, in studies of rats subjected to short-term high-fat feeding, excess fat has been shown to accumulate in the liver before adipose tissue [3,4].

In the absence of alcohol consumption, viral infection, or other specific etiologies, hepatic neutral lipid accumulation has been defined as nonalcoholic fatty liver disease (NAFLD) [5], which is the hepatic manifestation of metabolic syndromes such as obesity, diabetes, and hyperlipidemia [1]. Although multiple metabolic abnormalities may contribute to the development of fatty liver disease, insulin resistance is strongly associated with NAFLD [3,4,6]. Accordingly, NAFLD has become a major challenge to healthcare systems worldwide because of the increasing prevalence of its major risk factors, namely obesity and type 2 diabetes, which are closely linked to overeating, physical inactivity, and metabolic syndrome [7,8]. Therefore, an important consideration of current research should be whether salutary metabolic effects, such as prevention of hepatic lipid accumulation and improvement of lipid profile, could be produced by phytochemicals in food.

Numerous studies have suggested that natural compounds in food can be important modulators in the prevention of a variety of chronic diseases [9,10]. Increasing evidence suggests that anthocyanins are potent antioxidants, which are associated with protective effects observed against inflammation, atherosclerosis, carcinoma, and diabetes [11-17]. Anthocyanins are a class of water-soluble natural pigments that belong to the large family of flavonoids, responsible for the red, purple, and blue colors of many plant materials [18,19]. Cyanidin-3-glucoside (C3G) is the primary anthocyanin constituent in black rice [20], an important source of anthocyanins in Asia [21]. Recent studies have reported that treatment with purified C3G ameliorates adipose inflammation and hepatic steatosis in high-fat diet-fed mice [22], as well as hyperglycemia in diabetic mice [23,24]. In addition, black rice contains various bioactive phytochemicals, such as tocopherols, tocotrienols, oryzanols, vitamin B complex, and phenolic compounds [21,25].

We hypothesized that black rice containing C3G may reduce the risk of hepatic fat accumulation and improve insulin resistance in high-fat diet-fed mice and tested this hypothesis in this study. Furthermore, we assumed that crude black rice extract (BRE) may produce more potent effect than the purified compounds, as crude extracts can exert the additive or synergistic effects by the presence of multi phytochemicals [26]. Moreover, the mechanism by which anthocyanin treatment alleviates hepatic steatosis in vivo is not yet fully understood. In the liver, fatty acid oxidation occurs via mitochondrial and peroxisomal β-oxidation and microsomal CYP4A-catalyzed ϖ-oxidation [2,27-29]. The key enzymes involved in fatty acid oxidation systems, such as CPT1A, ACO, and CYP4A10, are principally regulated by peroxisome proliferator-activated receptor (PPAR)-α [2]. Therefore, to examine the mechanism underlying BRE inhibition of hepatic fat accumulation, we also measured the relative mRNA expression of PPAR-α, CPT1A, ACO, and CYP4A10 in the liver.

## Methods

### Ethical approval

This experimental design was approved by the Institutional Animal Care and Use Committee (IACUC) of the National Academy of Agricultural Science (reference number: NAAS-1114).

### Materials

The pigmented black rice (Heugjinjubyeo, *Oryza sativa L*.) was harvested in the experimental rice field of the National Institute of Crop Science, Rural Development Administration (Suwon, Korea, 2010). The rice seeds were dehulled, milled in a laboratory mill, and passed through a 60-mesh sieve. The powdered black rice was extracted by overnight shaking in a 10× volume of 80% ethanol solution at room temperature. The extract was filtered through No. 6 filter paper (Advantec; Dublin, CA, USA). The filtrates were concentrated into dry solids by sequential use of a rotary evaporator (EYELA; Tokyo, Japan) and lyophilizer (Ilshin Lab. Co.; Seoul, Korea). The final dried extract was stored at -20°C until use. The BRE yield was 2.24% on the basis of the fresh weight.

### Analysis of anthocyanin composition and content in black rice

**Figure 1:**
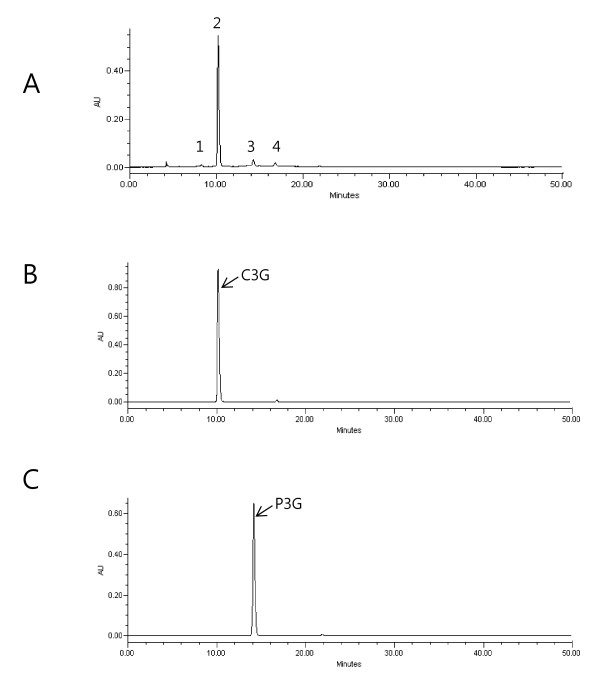
**Liquid chromatograms of anthocyanin extracted from BRE (A), C3G standard (B), and P3G standard (C)**. Peak 1, Cyanidin 3,5-diglucoside; Peak 2, Cyanidin-3-glucoside; Peak 3, peonidin-3-glucoside; Peak 4, unknown (cyanidin-based).

Total anthocyanin components of BRE were measured using liquid chromatography-mass spectrometry (LC-MS; Waters Co., Milford, MA, USA) by comparing their retention time and MS data with control standards (Extrasynthèse; Genay, France) and our previous data [30]. BRE (20 μL) was injected onto a Synergi Polar-RP 80A reversed-phase column (4.6 × 250 nm I.D.; 4 μm Phenomenex; Torrance, CA, USA). The analysis was conducted at a flow rate of 1 mL/min at a detection wavelength of 250-600 nm (a representative wavelength of 525 nm) and the oven temperature was 30°C. The mobile phases used were 5% formic acid in water (phase A) and 5% formic acid in water/acetonitrile (1:1, v/v) (phase B). The pretreated sample was analyzed using the following gradient conditions: a gradient of 20% to 50% B over a 30-min period; 50% B for 5 min; a gradient of 50% to 20% B over a 5-min period; followed by a final wash with 20% B for 10 min. Figure 1 shows the anthocyanin profile of BRE. The contents of C3G and peonidin-3-glucoside (P3G) in BRE were 23.21 mg/g and 1.69 mg/g, respectively. The total anthocyanin value was expressed as the sum of C3G and P3G equivalents per gram of BRE (mg/g). The total anthocyanin content was as high as 2.5% of the BRE. The proportion of C3G to total anthocyanin content was 93%. C3G was identified as the major peak in BRE.

### Animals and diet

Five-week-old male C57BL/6 J mice were purchased from Charles River Laboratories Japan, Inc (Tsukuba, Japan). Mice were housed in Plexiglas cages (2 to 3 mice per cage) and maintained in an air-conditioned room at 23 ± 3°C under an automatic lighting schedule. The mice were allowed free access to water and a laboratory diet for 10 d for acclimation. After the acclimation period, the mice were randomly blocked into three groups (n = 8 per group) with a similar mean body weights. The mice in one group were fed a normal diet (ND, 16.7% of calories derived from fat), while those in the second group were given a high-fat diet (HF, 45% of calories derived from fat), and the third group was fed a high-fat diet supplemented with 1% BRE (HF + BRE1%, HF with 1% BRE of diet weight). Finally, the C3G content of the 1% BRE diet was 0.023% (0.23 g/kg of diet). The compositions of the experimental diets are displayed in Table 1. The treatment period was 7 wk.

**Table 1:** Composition of the experimental diets*^1) ^*(in grams)

Ingredients	ND	HF	HF + BRE 1%
Cornstarch	397.49	266.5	256.5
Dextrin	132	88.5	88.5
Sucrose	100	67.1	67.1
Fiber	50	50	50
Casein	200	240.4	240.4
Corn oil	70	11.34	11.34
Beef tallow	-	215.46	215.46
Mineral Mix *^2)^*	35	42.1	42.1
Vitamin Mix *^2)^*	10	12	12
L-cysteine	3	3.6	3.6
Choline bitartrate	2.5	3	3
Tert-butylhydroquinone	0.014	0.017	0.017
Black rice extract Cyanidin-3-glucoside *^3)^*	-	-	10 (0.23)
Total amount (g)	1000.0	1000.0	1000.0

*^1) ^*Diets were prepared according to the AIN-93 G diet with slight modifications

*^2) ^*Mineral mixture and vitamin mixture were prepared according to AIN-93 G diet

^3) ^The content of C3G in black rice extract is based on LC/MS measurement

### Preparation of the blood and tissue samples

At the end of experimental period (7 wk), mice were sacrificed after a 15-h starvation period. Blood samples obtained by cardiac puncture were centrifuged at 2,000 rpm for 20 min at 4°C, and the serum supernatant was transferred to a new microtube and stored at -20°C prior to use. The mouse livers were removed, rinsed with cold phosphate-buffered saline, and then weighed. Tissues were cut into pieces in a consistent manner. Pieces to be used for histological evaluation were stored in 10% buffered neutral formalin, and the remaining pieces were placed in -70°C deep freezer.

### Biochemical measurement

Serum FFAs concentration was measured by enzymatic colorimetric methods (BioAssays Systems, Hayward, CA, USA). Triglyceride (TG), total cholesterol (TC), and HDL-cholesterol (HDL-C) concentrations were measured using a kit (Asan Pharmaceutical; Seoul, Korea) based on an enzymatic colorimetric method. All procedures were performed in accordance with the manufacturer's instructions. LDL-cholesterol (LDL-C) was calculated according to the following formula:

LDL‐cholesterol=Totalcholesterol-HDL-cholesterol-Triglyceride/5

Fasting glucose concentration was determined using a portable glucometer (LifeScan; Milpitas, CA, USA). Serum insulin concentration was measured using an enzyme-linked immunosorbent assay (ELISA) kit according to the manufacturer's instructions (Millipore; Bedford, MA, USA). The homeostasis model assessment (HOMA) is a method used to quantify insulin resistance and beta cell function [31]. The fasting blood glucose level and serum insulin concentration were used to calculate homeostasis model assessment-estimated insulin resistance (HOMA-IR). One international unit of insulin (1 IU) is defined as the biological equivalent of approximately 45.5 μg of pure crystalline insulin (precisely 1/22 mg). The following formula was used to calculate HOMA-IR:

$$\text{HOMA - IR}=\text{Fasting}\;\text{glucose}\left(\text{mg}/\text{dL}\right)\times \text{Fasting}\;\text{insulin}\left(\text{mU}/\text{L}\right)/405$$

### Histological analysis of liver

Standardized pieces of liver tissue was fixed in 10% formalin and embedded in paraffin. Sections were cut and stained with hematoxylin and eosin (H&E). Images were captured using an Olympus AX 70 camera (Japan). Hepatic steatosis was graded as 0 (fatty hepatocytes occupying < 5%), 1 (fatty hepatocytes occupying 5-33%), 2 (fatty hepatocytes occupying 34-66%), or 3 (fatty hepatocytes occupying > 66%) according to the percentage of hepatic lipid [32]. Initial assessment was performed under low magnification (40× to 200×) and confirmed under high magnification (400×).

### Determination of hepatic mRNA expression

Total RNA was isolated from the excised mouse livers using Trizol reagent (Invitrogen; Carlsbad, CA, USA) according to the manufacturer's recommendations. Single-stranded cDNA was synthesized from 2 μg of total RNA using the High Capacity RNA-to-cDNA Kit (Applied Biosystems; Foster City, CA, USA) according to the manufacturer's instructions. Real-time quantitative PCR was performed with a Step-One-Plus RT-PCR System (Applied Biosystems). Relative mRNA levels were measured using the system above via TaqMan analysis that employed gene-specific primers and probes. The oligonucleotide sequences of primers and probes for TaqMan analysis of PPAR-α (Assay ID Mm00440939_ml); CPT1A (Assay ID Mm00550438_ml); ACO (Assay ID Mm01246831_ml); cytochrome P450, family 4, subfamily a, polypeptide 10 (CYP4A10) (Assay ID Mm Mm01188913_g1); and beta-actin (Assay ID Mm00607939_sl) were all obtained from Applied Biosystems. The relative expression levels of each gene were determined by the 2^-ΔΔCt ^method. Cycling conditions were as follows: one denaturing cycle at 95°C for 10 min, followed by 40 cycles at 95°C for 15 sec, and 60°C for 1 min.

### Statistical analysis

Data were expressed as mean ± SE for each group. All statistical analyses were performed with SAS 9.2 (SAS Institute; Cary, NY, USA). Results were analyzed by one-way analysis of variance (ANOVA). When a significant difference was indicated, a Duncan's multiple-range test was performed to determine significant differences among the groups. A p value < 0.05 was considered statistically significant.

## Results

### Effect of BRE on body and liver weight

The initial and final body weights and relative liver weights are shown in Table 2. The initial and final body weights did not significantly differ among the three groups, indicating that consumption of a high-fat diet for 7 wk did not affect mouse body weight. The relative liver weight (per 100 g body weight) in the HF group was significantly higher than in the ND group (p < 0.01). However, the HF + BRE 1% group had a lower mean liver weight than the HF group (p < 0.01), suggesting that supplementation of a high-fat diet with the 1% BRE diet reduced liver weight. The food intake of high-fat diet groups tended to decrease compared with that of ND, but the calorie intake were similar for all groups (Additional file 1: Table S1).

**Table 2:** Body and liver weights of C57BL/6 J mice fed different diets for 7 weeks*^1)^*

Weight	ND	HF	HF + BRE 1%
Initial body (g)	20.88 ± 0.47 ^NS^	20.99 ± 0.36	20.13 ± 0.40
Final body (g)	32.65 ± 0.63 ^NS^	32.24 ± 0.36	34.20 ± 0.65
Relative liver (% BW)*^2)^*	3.80 ± 0.17^b^	4.48 ± 0.23^a^	3.18 ± 0.10^c^

*^1) ^*Values are expressed as mean ± standard error (n = 8 for each group). Within the same row, means with different letters (a, b, or c) are significantly different at *p *< 0.05 by Duncan's multiple range test. NS; not significant by ANOVA at *p *< 0.05

*^2) ^*Relative liver weight is represented per 100 g of body weight (BW)

### Effect of BRE on lipid profile and insulin resistance index

**Figure 2:**
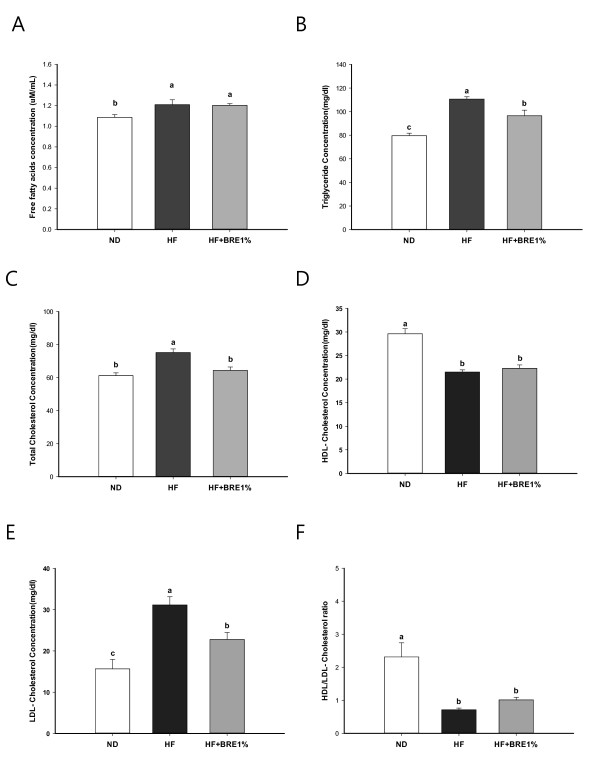
**Effect of BRE supplementation on serum lipid concentration**. Concentrations of serum FFAs (A), TG (B), TC (C), HDL- C (D), LDL-C (E), and HDL/LDL-C ratio (F) for each group during 7-wk feeding experiments. Data are expressed as mean ± standard error (n = 8 per group). Means with different letters (a, b, or c) on the bar are significantly different from each other at p < 0.05 by Duncan's multiple range test.

**Figure 3:**
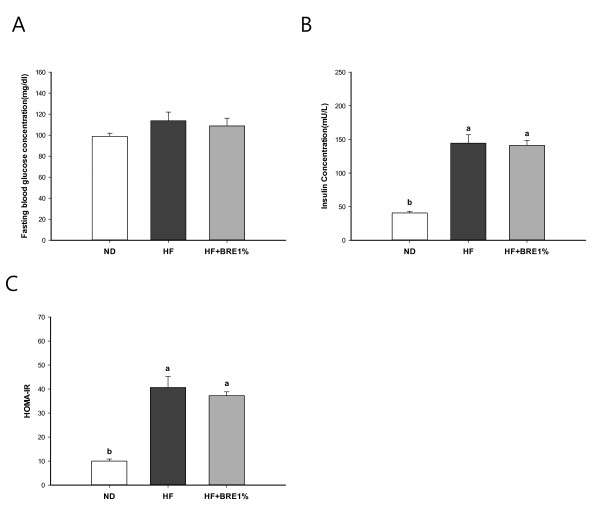
**Effect of BRE supplementation on blood concentration of glucose and insulin**. Concentrations of fasting blood glucose (A), and serum insulin (B), and insulin resistance index (C) for each group during 7-wk feeding experiments. HOMA-IR = fasting glucose (mg/dl) × fasting insulin (mU/L)/405. Data are expressed as mean ± standard error (n = 8 per group). Means with different letters (a, b, or c) on the bar are significantly different from each other at p < 0.05 by Duncan's multiple range test.

The serum basic biomarkers data are shown in Figure 2. The serum FFAs, TG, and TC levels of the HF group were significantly higher than those of the ND group (Figure 2; p < 0.01 for all). High-fat diet reduced HDL-C level and increased LDL-C level. Supplementation with 1% BRE effectively reduced serum TG, TC and LDL-C levels, but did not affect FFAs and HDL-C levels (Figure 2A). The serum glucose, insulin levels, and HOMA-IR of the HF + BRE1% group showed a trend towards reduction that approached, but did not attain, statistical significance compared to the HF group (Figure 3).

### Effect of BRE on hepatic steatosis

**Figure 4:**
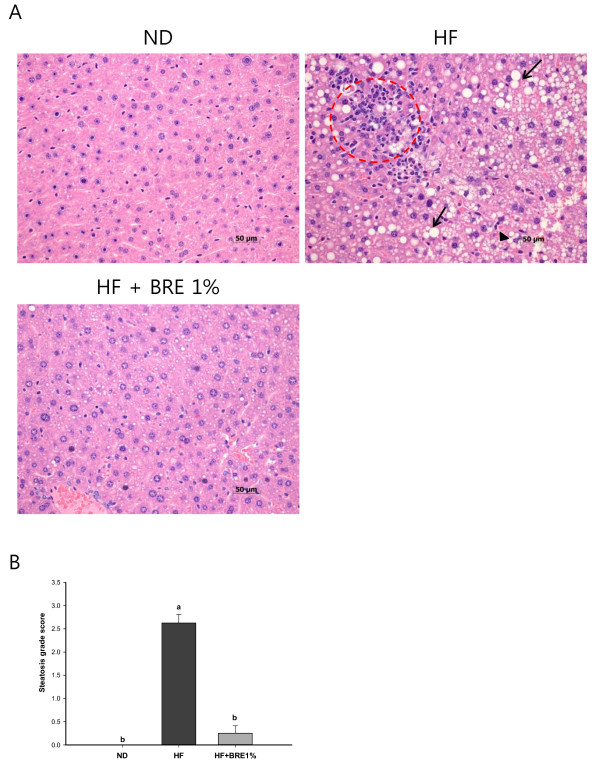
**Effect of BRE supplementation on liver histology**. Photomicrographs of H&E stained longitudinal liver sections from representative animals in the ND, HF, and HF + BRE1% groups (magnification 400×) (A). Lipid droplets (arrows), microvesicular steatosis (arrow head), and mild inflammation (circle) were observed in the HF liver sections. The HF group had a higher mean hepatic steatosis grade (0-3 scale) than the ND and HF + BRE1% groups (B). Data are expressed as means ± standard error (n = 8 per group). Means with different letters (a, b, or c) on their associated bars are significantly different from each other at p < 0.05 by Duncan's multiple range test.

To investigate the inhibitory effect of dietary BRE supplementation on hepatic fat accumulation, we analyzed the histology of liver tissue using H&E staining. Marked microvesicular steatosis accompanied by a partial mild inflammation was observed in the HF group, as shown in Figure 4B. On the other hand, the degree of hepatic fat accumulation was substantially alleviated by dietary intake of BRE, as indicated by the reduced surface area of steatosis observed in livers from mice in the HF + BRE1% group (Figure 4A). The average steatosis grade scores for the ND, HF, and HF + BRE1% groups were 0, 2.63, and 0.25, respectively (Figure 4B). The steatosis grade scores were significantly lower in the BRE groups than in the HF group (p < 0.01). We found that BRE supplementation was associated with a lower incidence of visible steatotic livers in the HF + BRE1% group compared to the HF group lacking BRE supplementation. Although, mice fed a high-fat diet for 7 wk did not become obese, they did exhibit hepatic fat accumulation similar to NAFLD. These observations are consistent with the expectation that excessive dietary fat accumulates in the liver before accumulating in adipose tissue under high-fat dietary conditions.

### Effect of BRE on gene expression involved in fatty acid β-oxidation in liver

**Figure 5:**
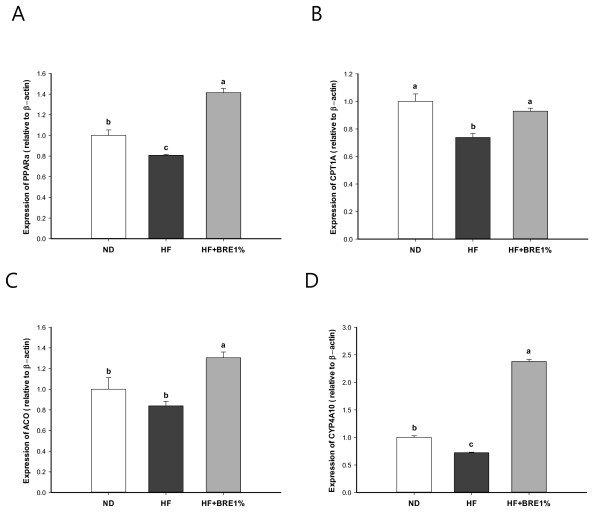
**Effect of BRE supplementation on mRNA levels of PPAR-α (A), CPT1A (B), ACO (C), and CYP4A10 (D) in liver of mice that were fed different diets**. Data are expressed as mean ± standard error (n = 8 per group). Means with different letters (a, b, or c) on the bar are significantly different from each other at p < 0.05 by Duncan's multiple range test.

Our measurements of relative mRNA expression of PPAR-α, CPT1A, ACO, and CYP4A10 in the liver by real-time quantitative PCR are summarized in Figure 5. The mRNA expression level of CPT1A, which is considered the rate-limiting enzyme in fatty acid oxidation, was decreased in the HF group (Figure 5B; p < 0.01). The mRNA expression levels for ACO and CYP4A10, involved in peroxisomal β-oxidation and microsomal ω-oxidation of fatty acids were also reduced in the HF group (Figure 5C and 5D; p < 0.05 and p < 0.01, respectively). On the other hand, dietary BRE supplementation significantly increased the mRNA expression levels of PPAR-α, CPT1A, ACO, and CYP4A10 (Figure 5; HF + BRE1% vs HD, p < 0.05 for all).

## Discussion

The primary outcome of this study was the finding that dietary BRE supplementation improved serum lipid profiles and attenuates hepatic steatosis in a high-fat diet mouse model of NAFLD. It is our expectation that these positive effects of BRE supplementation can likely be attributed to the C3G within our BRE; C3G is known to exert anti-oxidative and anti-inflammatory effects [15,16]. This finding is highly promising, suggesting that salutary effects such as those observed here may have the potential to reduce the risk of hepatic diseases, including NAFLD. High-fat diet induced the decrease of the activities of the antioxidant enzyme such as SOD and CAT in the liver [33]. It is believed that increased oxidative stress have an effect on the development of NAFLD [34].

In this study, we found that HF mice had increased serum levels of FFAs, TG, TC, and insulin, and an increased HOMA-IR index compared to ND mice. These findings fit with prior evidence indicating that higher insulin levels co-exist with elevated levels of TC and TG in the progressive state of NAFLD [35], as well as studies showing that suppression of very-low-density lipoprotein secretion and fatty acid β-oxidation produces hepatic TG accumulation [5,6]. Although body weight change did not differ among the groups in our study, we did observe a significant increase in relative liver weight and hepatic steatosis accompanied by a partial mild inflammationin the HF group. Related research has shown that a short-term high-fat causes hepatic steatosis in rats without any significant increase in visceral or skeletal muscle fat content [3,4].

We found that, in mice being fed a high-fat diet, dietary BRE supplementation significantly reduced serum levels of TG and TC and showed non-significant trends towards reducing serum levels of glucose and insulin and HOMA-IR. Although no significant effects of dietary BRE supplementation on insulin resistance were observed in our model, the reduced serum TG and TC levels observed in the HF + BRE1% group were associated with a reduction in hepatic steatosis relative to that seen in the HF group [35]. BRE supplementation was also associated with fewer visible steatotic livers compared with that in mice fed a high-fat diet without BRE supplementation. These results suggest that BRE-supplemented mice had lower risk of hyperlipidemia and were protected from reaching the progressive state of NAFLD.

Activation of AMP-activated protein kinase (AMPK) downregulates enzymes involved in gluconeogenesis, and subsequent activation of PPAR-α by AMPK decreases TG levels in the liver by stimulating fatty acid oxidation [36]. Tsuda *et al. *also suggested that AMPK activation by cyanidin may be linked to the expression of genes, including genes encoding adipokine and lipolysis enzymes [17]. Guo *et al. *reported that supplementation with C3G (0.2%) attenuated obesity-associated insulin resistance and hepatic steatosis in high-fat diet-fed mice [22].

Previous studies have shown that PPAR-α, together with the products of its target genes (i.e., CPT1A, ACO, and CYP4A10), is a critical player in the development of NAFLD [2,29]. Thus, in an effort to examine the underlying mechanisms of BRE inhibition of hepatic fat accumulation, we measured the relative mRNA expression levels of fatty acid metabolism-related genes, including PPAR-α, CPT1A, ACO, and CYP4A10 and found that the expression of these genes were reduced by a high-fat diet. These findings complement recent studies reporting that mRNA expression levels of CPT1A and ACO were reduced in animals fed high-fat diets for 7 ~ 12 wk [1,27]. It is worth noting that the mice fed BRE-supplemented high-fat diet in our study had significantly higher expression levels of PPAR-α, CPT1A, ACO, and CYP4A10 than did the HF control mice. CPT1A is considered the rate-limiting enzyme in mitochondrial fatty acid oxidation [27]. Microsomal ω-oxidation, which is mediated by cytochrome p450 enzymes, plays a pivotal role in energy homeostasis and lipid accumulation [37,38]. Long-chain fatty acids and very long-chain fatty acids are metabolized by the CYP4A ω-oxidation system into dicarboxylic acids that then serve as substrates in peroxisomal β-oxidation [39]. The upregulated PPAR-α-dependent peroxisomal beta-oxidation pathway induced in hepatic steatosis provides an alternative mechanism for removal excessive fatty acids from the tissue [40]. Thus, we propose that supplementary BRE may prevent hepatic steatosis induced by a high-fat diet by allowing increased fatty acid oxidation. The additional mechanism of inhibition of hepatic fat accumulation can be explained by a down-regulation lipogenic genes including SREBP-1c. König *et al. *reported that activation of PPAR-α reduces triacylglycerol synthesis in rat hepatoma cells by reduction of nuclear SREBP-1 [41]. Tsuda *et al. *showed that dietary C3G-rich purple corn suppressed the mRNA levels of enzymes involved in TG synthesis and lowed SREBP-1 mRNA level in the liver [42]. However, in this study, there was no significant difference in SREBP-1 gene expression of the liver among groups (Additional file 2: Figure S1).

The role of fatty acid oxidation in the liver is controversial. On one hand, fatty acid oxidation is viewed as a protective mechanism for the disposal of potentially toxic FFAs, but on the other hand, increased oxidation of fatty acids can generate reactive oxygen species (ROS), which cause tissue damage [40]. Peroxisomes also have anti-oxidant enzymes to prevent ROS-mediated cell damage [43]. Numerous *in vitro *and *in vivo *studies have reported that black rice possesses anti-oxidative effects [44-48]. Here, however, we did not examine liver ROS levels or antioxidant enzymes such as hepatic superoxide dismutase and catalase. It is noteworthy that oxidative stress-related inflammation was not apparent in the histology of our HF + BRE1% group, suggesting that the BRE supplementation was not associated with concerning levels of ROS-mediated cell damage. Nevertheless additional research will be needed to determine whether the positive effects of BRE on lipid metabolism and related antioxidant actions.

## Conclusions

Dietary BRE supplementation significantly enhanced mRNA expression levels of fatty acid metabolism-related genes in the liver, most notably genes related to β-oxidation and ω-oxidation. The enhancement of fatty acid oxidation was not associated with an accumulation of TG in the liver. These results suggest that black rice containing C3G is a healthful food that may be useful in the reducing the risks of hepatic steatosis and its related disorders such as hyperlipidemia and hyperglycemia.

## Abbreviations

FLD: fatty liver disease; TG: triglyceride; NAFLD: non alcoholic fatty liver disease; BRE: black rice extract; FFAs: free fatty acids; TC: total cholesterol; CPT: carnitine palmitoyltransferase; ACO: acyl-CoA oxidase; CYP4: cytochrome P450; PPAR: peroxisome proliferator activated receptor; C3G: cyanidin-3-glucoside; P3G: peonidin-3-glucoside; HOMA: homeostasis model assessment; AMPK: AMP-activated protein kinase.

## Competing interests

The authors declare that they have no competing interests.

## Authors' contributions

The author's responsibilities were as follows: HHJ performed the experiments and analysis and manuscript writing, MYP conducted the animal experiments and analysis, HWK analyzed the contents of anthocyanins, YML and KAH participated in data interpretation and reviewed the manuscript, JHP performed histological analysis, DSP and OK contributed to study design and manuscript writing. All authors read and approved the final manuscript.

## Supplementary Material

Additional file 1 **Table S1: Daily intakes of food and Calorie of C57BL/6J mice fed in different diets for 7 weeks**^1)^. ^1)^Value are expressed as mean ± standard error (n = 3 per group). NS; not significant by ANOVA at *p *< 0.05. ^2)^Daily food intake was determined base on the total weekly intake per cage (2-3 mice/cage). Click here for file

Additional file 2 **Figure S1: Effect of BRE supplementation on mRNA levels of SREBP-1 in liver of mice that were fed different diets**. Data are expressed as mean ± standard error (n = 8 per group). Not significant by ANOVA at *p *< 0.05. Click here for file
