# Supplementary material for: Black rice (Oryza sativa L.) extract attenuates hepatic steatosis in C57BL/6 J mice fed a high-fat diet via fatty acid oxidation
Source: Nutr Metab (Lond). 2012 Mar 30;9:27. doi: 10.1186/1743-7075-9-27 (PMC3366884; doi:10.1186/1743-7075-9-27)
Supplement: Additional file 1 — Table S1: Daily intakes of food and Calorie of C57BL/6J mice fed in different diets for 7 weeks1). 1)Value are expressed as mean ± standard error (n = 3 per group). NS; not significant by ANOVA at p < 0.05. 2)Daily food intake was determined base on the total weekly intake per cage (2-3 mice/cage). [file 1743-7075-9-27-S1.PPT]

## Slide 1
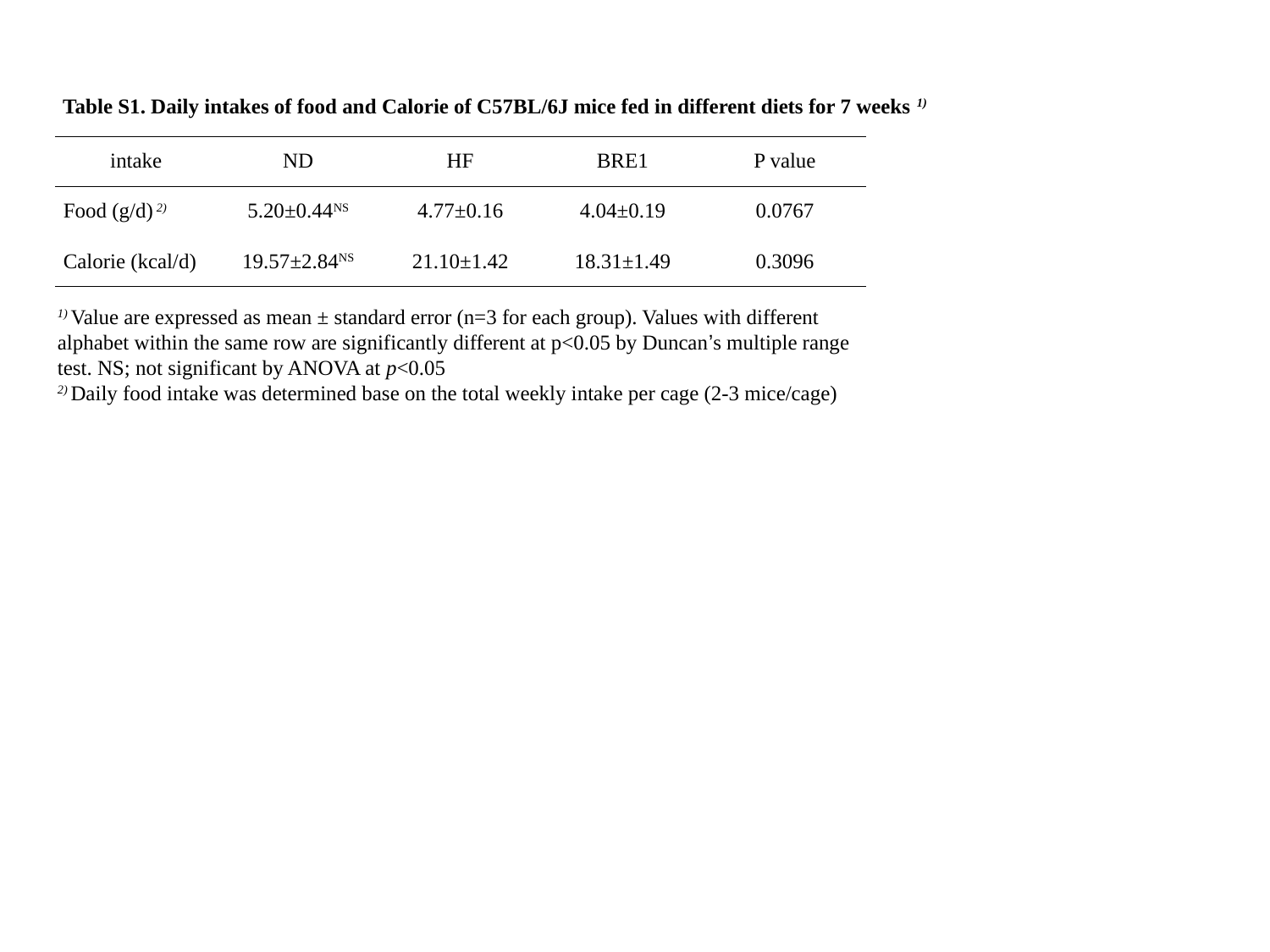

Table S1. Daily intakes of food and Calorie of C57BL/6J mice fed in different diets for 7 weeks 1)
| intake | ND | HF | BRE1 | P value |
| --- | --- | --- | --- | --- |
| Food (g/d) 2) | 5.20±0.44NS | 4.77±0.16 | 4.04±0.19 | 0.0767 |
| Calorie (kcal/d) | 19.57±2.84NS | 21.10±1.42 | 18.31±1.49 | 0.3096 |
1) Value are expressed as mean ± standard error (n=3 for each group). Values with different alphabet within the same row are significantly different at p<0.05 by Duncan’s multiple range test. NS; not significant by ANOVA at p<0.05
2) Daily food intake was determined base on the total weekly intake per cage (2-3 mice/cage)
