# Supplementary material for: Black rice (Oryza sativa L.) extract attenuates hepatic steatosis in C57BL/6 J mice fed a high-fat diet via fatty acid oxidation
Source: Nutr Metab (Lond). 2012 Mar 30;9:27. doi: 10.1186/1743-7075-9-27 (PMC3366884; doi:10.1186/1743-7075-9-27)
Supplement: Additional file 2 — Figure S1: Effect of BRE supplementation on mRNA levels of SREBP-1 in liver of mice that were fed different diets. Data are expressed as mean ± standard error (n = 8 per group). Not significant by ANOVA at p < 0.05. [file 1743-7075-9-27-S2.PPT]

## Slide 1
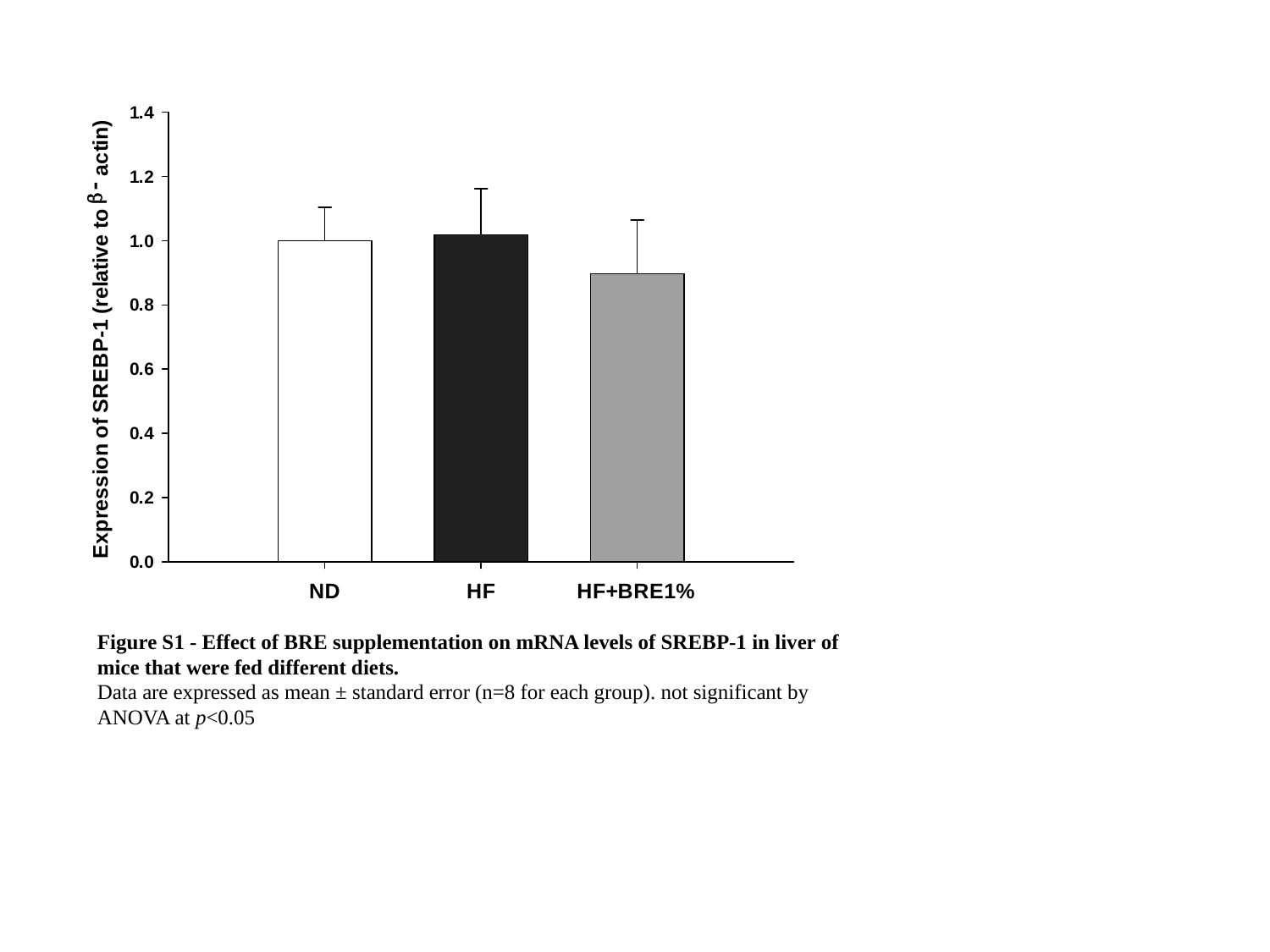

Figure S1 - Effect of BRE supplementation on mRNA levels of SREBP-1 in liver of mice that were fed different diets.
Data are expressed as mean ± standard error (n=8 for each group). not significant by ANOVA at p<0.05
